# Unraveling the Influence of *HHEX* Risk Polymorphism rs7923837 on Multiple Sclerosis Pathogenesis

**DOI:** 10.3390/ijms23147956

**Published:** 2022-07-19

**Authors:** Adela González-Jiménez, Pilar López-Cotarelo, Teresa Agudo-Jiménez, Marisa Martínez-Ginés, Jose Manuel García-Domínguez, Elena Urcelay, Laura Espino-Paisán

**Affiliations:** 1 Laboratorio de Genética de Enfermedades Complejas, Instituto de Investigación Sanitaria del Hospital Clínico San Carlos (IdISSC), 28040 Madrid, Spain; adela.gonzalez.jimenez@salud.madrid.org (A.G.-J.); pilar.lopezcotarelo@salud.madrid.org (P.L.-C.); tere844.taj@gmail.com (T.A.-J.); elena.urcelay@salud.madrid.org (E.U.); 2 Redes de Investigación Cooperativa Orientada a Resultados en Salud (RICORS), 28029 Madrid, Spain; 3 Servicio de Neurología, Hospital General Universitario Gregorio Marañón, 28007 Madrid, Spain; marisamgines@hotmail.com (M.M.-G.); jose_garciadom@hotmail.com (J.M.G.-D.)

**Keywords:** multiple sclerosis, HHEX, genetics, expression, metabolism

## Abstract

One of the multiple sclerosis (MS) risk polymorphisms, rs7923837, maps near the *HHEX* (hematopoietically-expressed homeobox) gene. This variant has also been associated with type 2 diabetes susceptibility and with triglyceride levels, suggesting its metabolic involvement. HHEX plays a relevant role as a negative regulator of inflammatory genes in microglia. A reciprocal repression was reported between HHEX and BCL6, another putative risk factor in MS. The present study evidenced statistically significant lower *HHEX* mRNA levels in lymphocytes of MS patients compared to those of controls, showing a similar trend in MS patients to the already described eQTL effect in blood from healthy individuals. Even though no differences were found in protein expression according to *HHEX* genotypes, statistically significant divergent subcellular distributions of HHEX appeared in patients and controls. The epistatic interaction detected between *BCL6* and *HHEX* MS-risk variants in healthy individuals was absent in patients, indicative of a perturbed reciprocal regulation in the latter. Lymphocytes from MS carriers of the homozygous mutant genotype exhibited a distinctive, more energetic profile, both in resting and activated conditions, and significantly increased glycolytic rates in resting conditions when compared to controls sharing the *HHEX* genotype. In contrast, significantly higher mitochondrial mass was evidenced in homozygous mutant controls.

## 1. Introduction

Multiple sclerosis (MS) is a chronic, inflammatory, demyelinating, immune-mediated disease that affects approximately 2.5 million people worldwide, with an increasing prevalence [1]. The clinical manifestations and course of MS are variable: in most patients, reversible episodes of neurological deficits characterize the initial phase of the disease (relapsing–remitting) and, over time, permanent neurological deficits and progression of clinical disability are developed (secondary progressive). Around 15% of patients show a progressive disease course from onset (primary progressive). MS typically debuts between 20 and 35 years old and it is the first cause of non-traumatic neurological disability in young adults. The etiology of the disease is still elusive, but the most accepted model proposes a combination of genetic and environmental factors [2]. For decades, the major histocompatibility complex (MHC) was the only genetic factor related to MS susceptibility. More recently, genome-wide association studies (GWAS) allowed the identification of 233 MS risk variants accounting for around 50% of the total MS heritability [3]. One of these MS-risk single nucleotide polymorphisms (SNPs), rs7923837, maps on chromosome 10q23.33, near the *HHEX* (haematopoietically-expressed homeobox) gene. The *HHEX* gene encodes an oligomeric protein that belongs to the homeobox protein family, mainly known for its role in embryonic development [4]. In fact, as it is a critical regulator of vertebrate development affecting different key pathways, *HHEX* knockout mice are not viable and die during mid-gestation [5]. Interestingly, *HHEX* null mice show cardiovascular, endocrine, liver, muscle, nervous system, and metabolic phenotypes, suggesting extensive multisystem roles for the protein product of this gene.

HHEX is a versatile protein that regulates cell activity through different mechanisms, including DNA distortion [6]. As a transcription factor, HHEX binds either to tandemly repeated recognition sequences or to other transcription factors, and its relevant role as a negative regulator of inflammation-related genes in microglia has been recently reported [7]. By inhibiting the eukaryotic translation initiation factor 4E (eIF4E)-dependent transport, it regulates the translocation of different mRNAs from the nucleus to the cytoplasm, and it is the first homeodomain protein that modulates mRNA transport independently of its role as a transcription factor [8]. In this sense, even though eIF4E is broadly expressed in all eukaryotic cell types, HHEX limits its activity, being a tissue-specific regulator that maintains expression only in myeloid cells, lung, thyroid, and liver in adults [9].

In the present study, we aimed to elucidate the influence on MS pathogenesis of the risk polymorphism located in the 3′-flanking region at 28 kb of the *HHEX* gene, rs7923837. A recently published work showed that this polymorphism acted as an eQTL (expression quantitative trait locus) for *HHEX* in blood samples from a cohort of healthy controls, with the minor allele decreasing *HHEX* expression levels [10]. Therefore, we not only tried to replicate the described eQTL effect of rs7923837 on the *HHEX* expression levels in healthy controls, but also to examine its role in MS patients. The integrative analysis combining GWAS and eQTL results is accepted to provide clues to pinpoint candidate genes for these complex conditions. Nonetheless, even the perfect colocalization between eQTL and GWAS signals does not establish causality, and functional approaches are ultimately required. Moreover, another proposed MS-risk factor, BCL6, has been shown to directly bind the *HHEX* locus, and a reciprocal repression was evidenced, as *BCL6* is upregulated in *HHEX*-deficient cells [11]. Therefore, we also aimed to investigate a potential epistatic effect between both genetic risk factors [12].

Given that HHEX is a key homeodomain transcription factor for the development of common lymphoid progenitor cells [13], and that an aberrant immune function underlies MS pathology [1], we pursued the study of peripheral blood mononuclear cells (PBMCs) as an accessible biological sample. Furthermore, an extensive bibliography has been published regarding the impact of the *HHEX* gene on type 2 diabetes susceptibility, even with the involvement of the exact MS-risk polymorphism already mentioned [14].

The studied *HHEX* polymorphism rs7923837 has been significantly associated with triglyceride levels by multiple linear regression analyses, and two other SNPs in the downstream region of the gene with total cholesterol levels [15]. Moreover, genome-wide association studies identified noncoding SNPs associated with type 2 diabetes and obesity in linkage disequilibrium (LD) blocks encompassing the *HHEX* gene [16,17,18]. These LD blocks contain highly conserved noncoding elements which overlap with the genomic regulatory blocks of the *HHEX* gene [19]. These results suggest the possible implication of HHEX in metabolic reactions and led us to explore the metabolic profile of immune cells isolated from MS patients and healthy controls.

Since 2007, GWAS studies have established associations between SNPs and disease risk. However, they lack the resolution needed to ascertain causal variants, because SNPs are usually found in linkage disequilibrium with multiple protein-coding loci as well as with non-coding gene-regulatory elements that act over long distances [20]. Even though comprehensive, multi-layered, and integrative approaches applying artificial intelligence workflows have recently been published [21], further work is needed to fully characterize putative effector genes. To deepen the understanding of MS pathogenesis, functional studies such as ours are warranted to prioritize causal genes. 

## 2. Results

### 2.1. Lymphocytes from MS Patients Show Lower HHEX Expression Than Those from Healthy Controls

**Figure 1:**
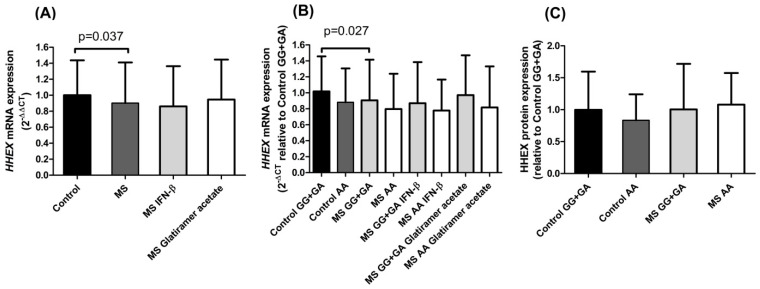
mRNA and protein expression of HHEX. (**A**) mRNA expression in controls, multiple sclerosis (MS) patients, and MS patients stratified by treatment. Controls: *n* = 117; MS patients: *n* = 154; MS patients treated with IFN-β: *n* = 94; MS patients treated with glatiramer acetate: *n* = 57. (**B**) *HHEX* mRNA expression in controls and MS patients stratified by rs7923837 genotype. Heterozygotes were grouped with major homozygotes due to similar expression levels. Control GG + GA: *n* = 101; minor allele homozygous controls AA: *n* = 16; MS GG + GA: *n* = 133; MS AA: *n* = 18; MS GG + GA treated with IFN-β: *n* = 85; MS AA treated with IFN-β: *n* = 9; MS GG + GA treated with glatiramer acetate: *n* = 48; MS AA treated with glatiramer acetate: *n* = 9. (**C**) Expression of HHEX protein analyzed by Western blot, stratified by rs7923837 genotype. Control GG + GA: *n* = 24; Control AA: *n* = 9; MS GG + GA: *n* = 36; MS AA: *n* = 13. Mean and standard deviation are presented in all panels.

Statistically significant lower *HHEX* mRNA levels were observed in PBMCs from MS patients compared to healthy controls (Figure 1A). As already mentioned, Ricaño-Ponce et al. [10] described that rs7923837 acts as an eQTL for *HHEX* in healthy controls. In accordance with these results, we found reduced levels of *HHEX* expression both in MS patients and controls carriers of the homozygous minor genotype rs7923837*AA (15% and 18%, respectively) when compared with carriers of the major allele (GG and GA), although these differences did not reach statistical significance (Figure 1B). Moreover, this tendency was consistently observed when MS patients were stratified according to treatment in both interferon-β and glatiramer acetate treated patients.

Regarding protein levels, the role of rs7923837 in controls followed a parallel situation to that found for mRNA, with a decreased protein expression in rs7923837*AA minor allele homozygotes. However, this trend was not evidenced in MS patients (Figure 1C).

### 2.2. Enriched HHEX Nuclear Localization in rs7923837*AA Homozygous MS Patients

**Figure 2:**
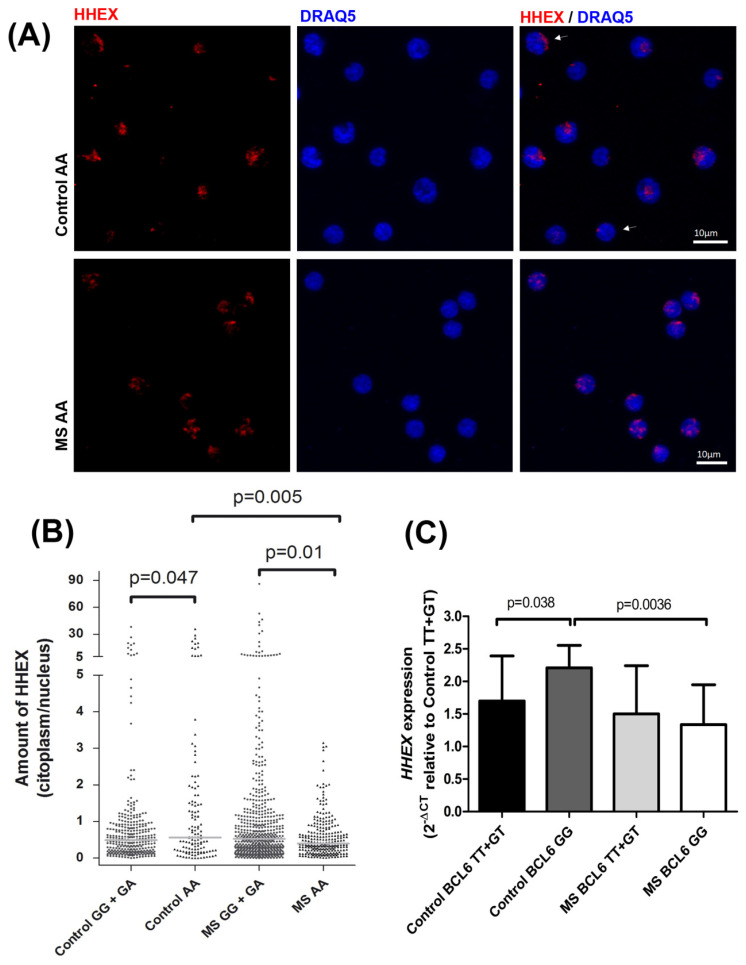
Subcellular localization of HHEX by confocal microscopy and epistatic interaction *HHEX*-*BCL6*. (**A**) Immunofluorescence of HHEX by confocal microscopy shows a higher localization of the transcription factor in the nucleus in rs7923837*AA multiple sclerosis (MS) patients. One confocal plane of each condition is shown. (**B**) Cytoplasmic/nuclear HHEX ratio measured by immunofluorescence and stratified by rs7923837 genotypes. Control carriers of the major allele GG + GA: *n* = 320 cells from 8 subjects; minor allele homozygous controls AA: *n* = 128 cells from 4 subjects; MS GG + GA: *n* = 599 cells from 17 subjects; and MS AA: *n* = 256 cells from 8 subjects. Red lines represent the median of the distribution (Control GG + GA median = 0.48; Control AA median = 0.56; MS GG + GA median = 0.52; MS AA median = 0.39). (**C**) *HHEX* mRNA expression stratified by the MS-risk polymorphism located near *BCL6*, rs2590438. Control carriers of the major allele TT + GT: *n* = 38; minor allele homozygous controls GG: *n* = 9; MS TT + GT: *n* = 40; and MS GG: *n* = 6. Mean and standard deviation are presented.

The activity of HHEX as a transcription factor can be linked to its cellular location. Thus, the influence of the *HHEX* polymorphism rs7923837 on nuclear translocation was analyzed by confocal microscopy, and a distinct effect of this SNP was observed in MS patients and in healthy controls (Figure 2A,B). Carriers of the major allele showed a higher nuclear location of HHEX than rs7923837*AA homozygous controls (*p* = 0.047). In contrast, the nuclear location of HHEX was significantly increased in rs7923837*AA homozygous patients when compared to MS carriers of the major allele (*p* = 0.01). Moreover, homozygous individuals for the risk genotype rs7923837*AA evidenced a significantly higher nuclear location of HHEX in MS patients compared to healthy controls (Control AA median = 0.56; MS AA median = 0.39, *p* = 0.005, Figure 2B).

As already mentioned, a reciprocal interaction between *BCL6* and *HHEX* has been reported [11]; therefore, we aimed to test whether the *HHEX* expression depends on the *BCL6* MS-risk polymorphism. To this end, the levels of expression of *HHEX* were stratified according to the *BCL6* rs2590438 genotypes (Figure 2C). A statistically significant increase was observed for the *BCL6* minor allele homozygous controls when compared to control carriers of the major allele (*p* = 0.004). In contrast, no difference was observed between MS subgroups, and a very significant difference was evidenced between minor allele homozygous patients and controls (*p* = 0.0036).

### 2.3. Mitochondrial Metabolism

The metabolic reprogramming of lymphocytes upon activation influences immune responses and ultimately affects disease progression. Consequently, we aimed to study the mitochondrial bioenergetics in the whole PBMC population, postulating that the crosstalk among the different immune subpopulations would determine the observed final outcome.

**Figure 3:**
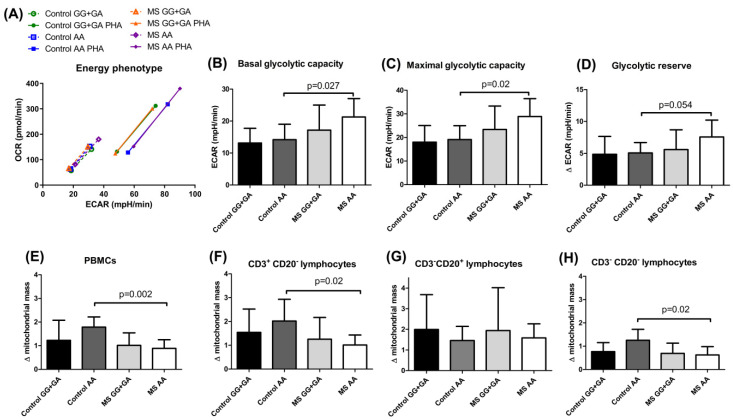
Glycolytic profile and mitochondrial mass of PBMCs from multiple sclerosis (MS) patients and controls stratified by the genotypes of HHEX rs7923837. (**A**) Energy phenotype of the studied groups. Minimum and maximum values of extracellular acidification rate (ECAR) and oxygen consumption rate (OCR) were confronted to obtain an estimation of the metabolic range of the cells. (**B**–**D**) Glycolytic analysis comparing carriers of the major allele in *HHEX* rs7923837 and minor-allele homozygotes from both MS patients and controls. Control GG + GA: *n* = 11; Control AA: *n* = 7; MS GG + GA: *n* = 18; MS AA: *n* = 7. (**E**–**H**) Increase in mitochondrial mass measured by flow cytometry with Mitotracker Green^TM^ after activation with PHA. The ratio of the median fluorescence intensity in the presence/absence of PHA was calculated for PBMCs (**E**) and the indicated lymphocyte subpopulations (**F**–**H**). Control GG + GA: *n* = 9; Control AA: *n* = 5; MS GG + GA: *n* = 17; MS AA: *n* = 8. Mean and standard deviation are presented in panels (**B**–**H**).

Upon PHA activation, immune cells increase their metabolic demands and, in our experimental conditions, the MS homozygotes for the rs7923837*AA genotype showed the more energetic phenotype (Figure 3A). When patients and controls stratified by *HHEX* genotypes were compared (Figure 3B–D), significant differences were consistently unrevealed for the Control vs. MS AA subgroups in basal and maximal glycolytic capacities and in glycolytic reserve in resting conditions. In addition, the increment in mitochondrial mass after PHA stimulation pinpointed homozygous controls for the rs7923837*AA genotype, with significantly higher mass in global PBMCs (Figure 3E), in the CD3^+^ (Figure 3F) and CD3^−^ CD20^−^ (mainly NK cells, Figure 3H) subpopulations, but not in B cells, which evidenced a similar pattern in MS patients and controls (Figure 3G).

## 3. Discussion

Efforts to unveil the underlying causal genes and mechanisms involved in complex diseases are demanded. The present study is an attempt to refine the role of one of the described MS-risk variants near the *HHEX* gene, rs7923837.

Our results regarding *HHEX* mRNA expression in PBMCs showed significantly lower levels in MS patients than in controls (Figure 1A). As reported, this *HHEX* polymorphism acts as an eQTL for the *HHEX* gene in blood samples of healthy subjects [10]. In accordance with this, in our cohorts, both controls and MS patients pointed to the previously described eQTL trend: the homozygous mutant genotype rs7923837*AA diminished *HHEX* expression (Figure 1B). Cell type-specific transcriptomic and epigenomic maps aid in the interpretation of the potential regulatory impact of rs7923837. The information provided by the Roadmap Epigenomics Project (roadmapepigenomics.org, accessed on 17 May 2022) revealed that this variant maps in a region that presents low transcriptional activity in PBMCs, as shown by high levels of repressive H3K27me3 histone methylation and low levels of H3K36me3 (found in areas undergoing active transcription).

Since no differences were found in HHEX protein expression levels associated with the rs7923837 genotypes (Figure 1C), we aimed to analyze the subcellular location of HHEX by confocal microscopy (Figure 2A). Both HHEX activities, as a transcription factor and as a suppressor of eIF4E-mediated mRNA transport, take place in the nucleus and, therefore, a higher nuclear location is indirectly related to its activity. Homozygotes for the rs7923837*AA genotype displayed a significantly different cellular distribution of HHEX when compared to carriers of the major G allele, both in MS patients (*p* = 0.01) and controls (*p* = 0.047) (Figure 2B). Moreover, while carriers of the *HHEX* major allele maintain a similar subcellular distribution in MS patients and controls, rs7923837*AA homozygotes showed an increased level of cytoplasmic HHEX in the control population, but an increment in nuclear HHEX in MS patients (*p* = 0.005).

Considering the described interaction of BCL6–HHEX, we observed significantly different levels of *HHEX* expression between the subgroups of controls stratified by the *BCL6* MS-risk polymorphism. This difference was lacking between the MS counterparts (Figure 2C), suggesting a defective reciprocal regulation, which could have an impact on the disease pathogenesis. This *BCL6*–*HHEX* epistatic interaction does not exhaust other possible epistatic effects, i.e., *HHEX* knockdown considerably enhanced the expression level of eomesodermin (EOMES), another MS-risk factor identified by GWAS [3]. HHEX binds to the HHEX-response element located in the first intron of the *EOMES* gene [22], indicative of the intricate network lying behind these complex diseases.

Emerging interest in metabolic reprogramming and its impact on lymphocyte activation led us to study the cellular bioenergetics in our cohorts stratified by the *HHEX* polymorphism with an already documented influence on metabolism. The immune system comprises a series of specialized cells able to rapidly respond to pathogens or inflammatory stimulus. Understanding how the metabolic activity influences immune responses and affects disease progression is of utmost importance in immune-mediated conditions such as MS. The bioenergetic profiling of lymphocytes has revealed that the cellular metabolism changes dynamically with activation. Upon antigen encounter, T cells undergo extensive proliferation and switch to a program of anabolic growth and biomass accumulation, with increased demand for ATP and metabolic resources. It has been described that HHEX is a key regulator of early lymphoid development and functioning [23]. Regulatory T cells (Tregs) play an essential role in maintaining the immune homeostasis and Tregs show lower expression of *HHEX* than conventional T cells [23]. As reported, HHEX directly binds to the promoters of Treg signature genes, such as *Foxp3*, *Il2ra*, and *Ctla4*, suggesting a role of HHEX as a Treg negative regulator. Specifically, the activity of the *Foxp3* promoter is almost completely inhibited by HHEX binding, and *Foxp3*, *Il2ra*, and *Ctla4* act as Treg-specific super-enhancers, which could easily be targets of the same transcription factor.

Regulating energy metabolism provides a way for T cells to reversibly switch between quiescent and highly proliferative states. In our experimental conditions, the MS carriers of the *HHEX* homozygous mutant genotype showed a distinctive, more energetic profile, both in resting and PHA-activated conditions (Figure 3A), and significantly increased glycolytic rates before PHA stimulation when compared to controls sharing the *HHEX* genotype (Figure 3B–D). Most probably, the continuous exposure to the self-antigen(s) would be responsible for the observed pre-activated glycolytic engagement in MS patients. It is important to remember that aerobic glycolysis is the dominant pathway in effector T cells, while resting naïve T cells maintain low rates of glycolysis and predominantly oxidize glucose-derived pyruvate via oxidative phosphorylation (OXPHOS) or use fatty acid oxidation (FAO) for ATP production. This strong bias toward glycolysis over mitochondrial metabolism was also evidenced by the significantly higher increase in mitochondrial mass upon PHA activation in homozygous mutant controls as compared to MS patients with the same genotype (Figure 3E,F,H), which did not seem to affect peripheral B cells (Figure 3G). Interestingly, integrated single-cell transcriptomics has recently revealed strong germinal center (GC)-associated etiology of autoimmune risk loci. In fact, many genetic variants implicated in autoimmunity exhibit their greatest regulatory potential in GC-associated cellular populations, including BCL6 and transcription factors regulating B cell differentiation, such as POU domain class 2 homeobox associating factor 1 (POU2AF1) and HHEX [24]. The role of HHEX seems confined to the generation of GC-derived memory, and it is not involved in the maintenance or function of memory B cells [25]. The key role of GC in both adaptive immunity and peripheral tolerance by limiting autoreactive B cells explains why dysfunction in these processes can lead to defective immune responses and autoimmune diseases. These compartmentalized studies provide a complementary perspective to our approach, which seeks to evaluate the comprehensive lymphocytic outcome resulting from the immune cellular crosstalk.

Furthermore, in the adult central nervous system (CNS), neurons possess a limited capacity to regenerate injured axons, limiting repair. In fact, activating pro-regenerative gene expression in CNS neurons is a promising therapeutic approach. HHEX is widely expressed in adult CNS neurons, but is present only in trace amounts in immature cortical neurons and adult peripheral neurons. HHEX overexpression in early postnatal cortical neurons reduced both initial axonogenesis and the rate of axon elongation, suggesting a role for HHEX in restricting axon growth in the developing CNS [26]. As mentioned, HHEX negatively regulates inflammation-related genes in microglia, opening provocative therapeutic avenues [7]. Altogether, these results provide clues for the interpretation of the genetic causes underpinning MS disease. Our work demonstrates how the *HHEX* genetic variant influences the subcellular location of the encoded protein involved in critical immune regulatory and metabolic profiles. Understanding the genetic bases of immune system regulation may have broad implications in the disease treatment.

## 4. Materials and Methods

### 4.1. Study Population: Patients and Controls

The study included a total of 154 MS patients (59.5% females) and 117 healthy controls (67.5% females), with mean ages of 40.9 ± 11.3 and 41.4 ± 10.1 years, respectively. Patients were diagnosed with relapsing remitting multiple sclerosis established according to McDonald’s criteria [27] which requires dissemination of lesions in space and time, resulting in an earlier diagnosis of MS with a high degree of both specificity and sensitivity. All patients were recruited from collaborating hospitals in the Madrid region during routine visits with their Neurology departments. Patients were treated with interferon-β formulations or glatiramer acetate and had no evidence of relapse before or after extraction. None of the control subjects reported first- or second-degree relatives with any immune-mediated disease. All participants were recruited after written informed consent, and the study was approved by the Ethics Committee from Hospital Clínico San Carlos.

Peripheral blood samples were collected and PBMCs (peripheral blood mononuclear cells) were separated with Lymphoprep density-gradient centrifugation following the manufacturer’s instructions (07851, Stemcell Technologies, Vancouver, BC, Canada); genomic DNA was extracted from the granulocyte phase following a salting-out procedure and was quantified and preserved at −20 °C; and PBMCs were cryopreserved in liquid nitrogen until further analysis.

### 4.2. Genotyping

Genotyping was achieved in 154 MS patients and 117 healthy controls by TaqMan technology on a 7900HT Fast Real-Time PCR System (Applied Biosystems, Foster City, CA, USA) following the manufacturer’s protocols. TaqMan probes for *HHEX* rs7923837 (C__31982553_10) and *BCL6* rs2590438 (C___1699097_10) were purchased from Applied Biosystems (Foster City, CA, USA). 

### 4.3. Gene Expression

Total RNA was isolated from the PBMCs of 154 MS patients and 117 controls with Trizol reagent following the manufacturer’s protocol (15596018, Invitrogen, Waltham, MA, USA). Samples were subsequently quantified in a NanoDrop ND-1000 spectrophotometer (NanoDrop Products; Wilmington, DE, USA) and reverse transcribed by using a High Capacity RNA-to-cDNA kit (4387406, Applied Biosystems, Waltham, MA, USA). cDNA was amplified by qPCR using TaqMan probes for *HHEX* (Hs00242160_m) and *GUSB* (4326320E) as a housekeeping gene on a 7900HT Fast Real Time PCR System (Applied Biosystems, Waltham, MA, USA). Data were analyzed with DataAssist v3.01 software (Applied Biosystems, Waltham, MA, USA). 

### 4.4. Western Blot

PBMCs (5 × 10^5^) of 49 MS patients and 33 controls were resuspended in 10 μL of SDS-PAGE sample buffer (100 mMTris/HCl, pH 6.8, 3% SDS, 1 mM EDTA, 2% 2-β-mercaptoethanol, 5% glycerol) and analyzed by Western blot. Briefly, PVDF membranes were incubated with anti-tubulin (600-401-880, Tebubio) and anti-HHEX (ab79392, Abcam, Cambridge, United Kingdom) primary antibodies, followed by anti-rabbit HRP-conjugated secondary antibody (A6154, Sigma Aldrich, Bremen, Germany), and visualized with an ECL substrate detection system (1705060, Biorad, Hercules, CA, USA). Blot quantification was performed using Fiji software [28].

### 4.5. Confocal Microscopy

PBMCs (2.5 × 10^5^) of 25 MS patients and 12 controls were plated for 1 h at 37 °C on dishes coated with polyornithine (20 μg/mL, P4957, Sigma Aldrich, Bremen, Germany) and fixed for 15 min with 4% paraformaldehyde at room temperature. Cells were stained with rabbit anti-human HHEX (HPA055460, Atlas Antibodies, Bromma, Sweden) as primary antibody, and a combination of biotinylated anti-rabbit antibody (BA-1000, Vector Laboratories, Newark, CA, USA) and streptavidin-Alexa Fluor 555 (S32355, Invitrogen, Waltham, MA, USA) supplemented with Draq5 (ab108410, Abcam, Cambridge, United Kingdom). Coverslips were mounted in fluorescent mounting medium (DAKO) and photographed with an Olympus FV3000 Confocal Laser Scanning Microscope. Images were quantified with Fiji software, an open-source image processing application [28]. In order to quantify the relative amount of nuclear HHEX compared to the cytoplasmic, the raw integrated intensity of the fluorescence signal was calculated for the whole cell, and another was calculated for the area in the cell overlapping the nuclear dye Draq5. The cytoplasmic HHEX signal was calculated by subtracting the nuclear signal (Draq5 + HHEX) from the whole cell signal. Finally, the ratio between the nuclear and cytoplasmic signal was calculated. 

### 4.6. Metabolic Analysis

A total of 25 MS patients and 18 controls were included. Oxygen consumption rate (OCR) and extracellular acidification rate (ECAR) of 2 × 10^5^ PBMCs were simultaneously analyzed in a Seahorse XFp extracellular flux analyzer (Agilent, Santa Clara, CA, USA). Cells were either left unstimulated or stimulated for 24 h with 5 μg/mL of PHA, and subsequently adhered onto poly-D-lysine (A38904-01, Gibco, Waltham, MA, USA) coated XFp plate wells for 45 min in XF DMEM medium, pH 7.4 (103575-100, Agilent Technologies, Santa Clara, CA, USA), supplemented with glucose (10 mM) and pyruvate (2 mM). Each condition was analyzed in triplicate with a Cell Mito Stress Test Kit (103010-100, Agilent Technologies, Santa Clara, CA, USA), according to the manufacturer’s instructions. Briefly, OCR and ECAR were measured in basal conditions and after oligomycin (1.5 μM), carbonylcyanide-p-trifluoromethoxyphenylhydrazone (FCCP.0.5 μM), and a cocktail of rotenone and antimycin A (both at 0.5 μM) injections.

### 4.7. Flow Cytometry

Excess PBMCs from the metabolic assay were stained the same day with Mitotracker Green (M46750, Invitrogen, Waltham, MA, USA) following the manufacturer’s instructions. Pairs of unstimulated and PHA stimulated samples (25 MS patients and 14 controls) were subsequently stained with anti-CD3-PE (clone HIT3a) and anti-CD20-APC (clone 2H7) to determine lymphocyte subpopulations and 7-AAD to exclude non-viable cells (Biolegend, San Diego, CA, USA) and were acquired immediately in a CytoFLEX cytometer (Beckman Coulter, Brea, CA, USA). Cells were gated according to surface markers: CD3^+^ CD20^−^ (T lymphocytes), CD3^−^ CD20^+^ (B lymphocytes), and CD3^−^ CD20^−^ (mostly NK cells). Mitotracker Green is a fluorescent dye that binds the mitochondria, so the amount of green fluorescent signal in each cell is correlated with the mitochondrial mass of the cell. Therefore, the median fluorescence intensity (MFI) of the Mitotracker Green signal was measured in every experimental group and for each lymphocyte subpopulation. The ratio of the MFI signal between PHA stimulated and unstimulated sample pairs was calculated to quantify the increase in mitochondrial mass after cell activation. Data were analyzed with Kaluza 2.1 software (Beckman Coulter, Brea, CA, USA).

### 4.8. Statistical Analysis

Normality was assessed with the Shapiro–Wilk test for datasets with less than 30 data and with the Kolmogorov–Smirnov test for datasets with 30 or more entries. Outliers were detected and discarded with Grubbs’ test (GraphPad online tool, https://www.graphpad.com/quickcalcs/Grubbs1.cfm, accessed on 1 April 2022). In analyses that conformed to the normal distribution, comparisons were performed with a Student’s *t*-test and ANOVA test, with Welch’s correction for samples with unequal variances. Those variables that did not follow a normal distribution were compared with Mann–Whitney U and Kruskal–Wallis tests. A standard *p* value of 0.05 was set for significance in all cases. Statistical analyses were performed with SPSS v15.0.1 (Chicago, IL, USA) and graphical representations were carried out with GraphPad v5.01 (San Diego, CA, USA). 

## Data Availability

The data that support the findings of this study are available upon request from the corresponding author.
